# Imputation of time-series scRNA-seq data with a time-varying blue noise diffusion model

**DOI:** 10.1093/bib/bbaf631.037

**Published:** 2025-12-12

**Authors:** Graham Bishop, Tong Si, Haijun Gong

**Affiliations:** Department of Mathematics and Statistics, Saint Louis University, St. Louis, MO, 63103, United States; Department of Health and Clinical Outcomes Research, Saint Louis University, St. Louis, MO, 63104, United States; Department of Mathematics and Statistics, Saint Louis University, St. Louis, MO, 63103, United States

## Abstract

Single-cell RNA sequencing (scRNA-seq) enables gene expression profiling at single-cell resolution, providing unprecedented insights into cellular heterogeneity and dynamic biological processes. When collected over time, time-series scRNA-seq data further capture temporal dynamics, allowing the reconstruction of cell type–specific gene regulatory networks and investigation of evolving transcriptional programs. However, scRNA-seq data are inherently sparse due to technical dropouts during sequencing, and this sparsity is amplified in time-series experiments. Such excessive sparsity undermines the reliability of downstream analyses, leading to biased characterization of disease trajectories and inaccurate inference of regulatory interactions. Numerous computational techniques have been proposed for missing value imputation.

Recent advances in generative models, such as generative adversarial networks and diffusion models, have achieved state-of-the-art performance in imputing missing values compared to traditional methods. However, existing approaches typically use white noise as the training prior [1], which fails to capture the frequency-dependent correlations and temporal structures intrinsic to biological time-series data. To address the limitations of traditional methods, our recent work [2] introduced a novel time-varying blue-noise–based conditional score diffusion model (tBN-CSDI) to impute missing values in time-series scRNA-seq data. We integrate Ulichney’s void-and-cluster algorithm with a Cholesky decomposition-based sampling strategy to generate blue noise. The proposed tBN-CSDI framework employs a frequency-aware noise schedule that interpolates between white and blue noise over time, such that high-frequency components are emphasized during the early stages of the reverse diffusion process and gradually transition toward lower frequencies as the process proceeds. This design enables the model to capture subtle, high-frequency temporal patterns while preserving global expression trends, thereby enhancing the accuracy of imputation. Experiments on two scRNA-seq datasets demonstrate that tBN-CSDI outperforms all existing imputation methods, significantly reducing the imputation error compared with other state-of-the-art approaches. This work was supported by the National Institutes of Health under award number R15GM148915.

**References:**

[1] Tashiro Y., Song J., Song Y., Ermon S. ‘CSDI: Conditional score-based diffusion models for probabilistic time series imputation.’ Advances in neural information processing systems 2021; 6; 34:24804–16.

[2] Bishop G., Si T., Luebbert I., Al-Hammadi N., Gong H.. ‘tBN-CSDI: a time-varying blue noise-based diffusion model for time series imputation.’ Bioinformatics Advances 2025; Volume 5, Issue 1, vbaf225.

